# Potent neutralizing human monoclonal antibodies protect from Rift Valley fever encephalitis

**DOI:** 10.1172/jci.insight.180151

**Published:** 2024-08-01

**Authors:** Kaleigh A. Connors, Nathaniel S. Chapman, Cynthia M. McMillen, Ryan M. Hoehl, Jackson J. McGaughey, Zachary D. Frey, Morgan Midgett, Connor Williams, Douglas S. Reed, James E. Crowe, Amy L. Hartman

**Affiliations:** 1 Department of Infectious Diseases and Microbiology, School of Public Health, and; 2 Center for Vaccine Research, University of Pittsburgh, Pittsburgh, Pennsylvania, USA.; 3 Department of Pathology, Microbiology and Immunology, and; 4 Vanderbilt Vaccine Center, Vanderbilt University Medical Center, Nashville, Tennessee, USA.; 5 Department of Immunology, University of Pittsburgh School of Medicine, Pittsburgh, Pennsylvania, USA.; 6 Department of Pediatrics, Vanderbilt University Medical Center, Nashville, Tennessee, USA.

**Keywords:** Immunology, Infectious disease, Adaptive immunity, Immunoglobulins, Neurological disorders

## Abstract

Rift Valley fever (RVF) is an emerging arboviral disease affecting both humans and livestock. In humans, RVF displays a spectrum of clinical manifestations, including encephalitis. To date, there are no FDA-approved vaccines or therapeutics for human use, although several are in preclinical development. Few small-animal models of RVF encephalitis exist, further complicating countermeasure assessment. Human mAbs RVFV-140, RVFV-268, and RVFV-379 are recombinant potently neutralizing antibodies that prevent infection by binding the RVFV surface glycoproteins. Previous studies showed that both RVFV-268 and RVFV-140 improve survival in a lethal mouse model of disease, and RVFV-268 has prevented vertical transmission in a pregnant rat model of infection. Despite these successes, evaluation of mAbs in the context of brain disease has been limited. This is the first study to our knowledge to assess neutralizing antibodies for prevention of RVF neurologic disease using a rat model. Administration of RVFV-140, RVFV-268, or RVFV-379 24 hours prior to aerosol exposure to the virulent ZH501 strain of RVFV resulted in substantially enhanced survival and lack of neurological signs of disease. These results using a stringent and highly lethal aerosol infection model support the potential use of human mAbs to prevent the development of RVF encephalitis.

## Abstract 2

This report shows that three human monoclonal antibodies recognizing Rift Valley fever (RVF) virus improve survival in an aerosol exposure rat model of RVF encephalitis.

## Graphical abstract

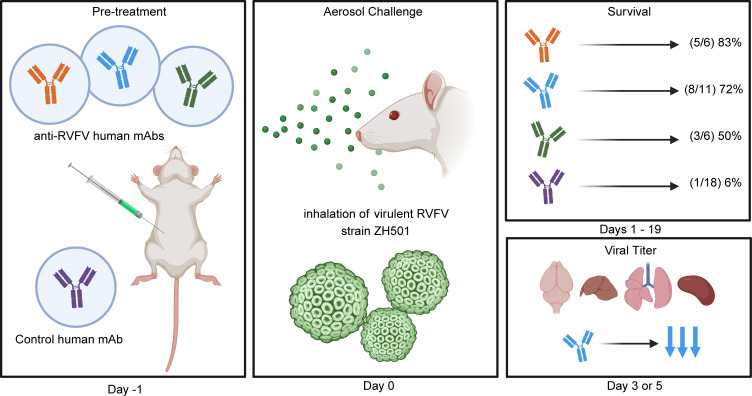

## Introduction

Prevention and treatment of infections caused by arboviruses present unique challenges given the sporadic nature and range of potential clinical outcomes that can occur. Rift Valley fever virus (RVFV) is an emerging bunyavirus that is considered a priority pathogen by the World Health Organization because of its epidemic potential (1). RVFV infects livestock and humans in Africa, the Middle East, and nations in the western Indian Ocean region (2, 3). Although humans typically develop a self-limiting febrile illness, 1%–5% of cases develop more severe disease outcomes, including hemorrhagic fever, encephalitis, ocular disease, and in utero infection during pregnancy. An array of clinical manifestations can be present in individual patients, which may include CNS involvement (4). Encephalitic disease following RVFV infection is rare, but it is associated with a 50% case fatality rate, and individuals who display neurologic symptoms may develop long-term neurologic sequelae (5). No FDA-approved human vaccines or therapeutics for RVF exist, although several are in the developmental pipeline (6–8). Importantly, none have yet addressed the need to prevent CNS infection and the neurological issues that can result.

One reason that Rift Valley fever (RVF) neurological disease countermeasures have remained unaddressed is that, until recently, mouse models of RVFV encephalitis have been limiting, as most inbred mouse strains succumb to lethal hepatic disease rapidly following inoculation with wild-type strains of RVFV (9). As an alternative to mice, our lab established a model in Lewis rats, whereby exposure of adult rats to inhaled RVFV results in development of lethal encephalitis within 7–10 days after infection (10). When rats are exposed through inhalation, RVFV travels across the olfactory epithelium and into the brain where it replicates to high levels and causes tissue damage (11). Infection, however, is not limited to the brain, and virus can be found in the liver, lung, spleen, and eye after inhalational exposure (12–14). Animals that succumb to RVFV infection via aerosol reproducibly display neurologic symptoms, including loss of muscle coordination/erratic movements, head tremors, circling, paralysis, and seizures.

mAbs, or combinations thereof, have been successful in the treatment of other emerging viral diseases (15, 16). Given that neutralizing antibody responses to RVFV are a primary correlate of protection and clearance (17), mAbs may be an effective path forward as a prevention and/or treatment modality for RVFV. Recently, Chapman et al. isolated a panel of human mAbs with potent neutralization capacity in vitro (Table 1) and demonstrated that several of the mAbs provided protection against subcutaneous RVFV infection in a lethal mouse model of hepatic disease (18). Two of these antibodies, designated RVFV-268 and RVFV-140, provide near 100% protection to mice when given 2 hours prior to, and up to 2 days after, a lethal dose of RVFV administered subcutaneously (18) and are thus remarkably able to effectively prevent lethal hepatic disease. While no synergy has been found, RVFV-268 and RVFV-140 reduced lethality at very low doses in mice when used as a combination therapy (19). A third antibody, RVFV-379, is a potent inhibitor of infection in vitro but has not yet been assessed in vivo.

The antibodies isolated by Chapman et al. primarily target the RVFV envelope glycoproteins Gn, Gc, or a combination of the 2 (18, 19). Epitope mapping studies show that RVFV-268 and RVFV-379 bind to monomeric Gn and bivalently bind an epitope accessible on the virion surface. RVFV-140, on the other hand, has an unmapped binding site on Gn/Gc, functions in mono- and bivalent formats, and acts as a fusion inhibitor (18). Here, we tested the ability of these human mAbs to prevent CNS infection and neurological disease using a stringent aerosol exposure model in rats.

## Results

### Monotherapy with human mAbs significantly improves survival following lethal inhalational exposure to pathogenic RVF virus.

**Figure 1:**
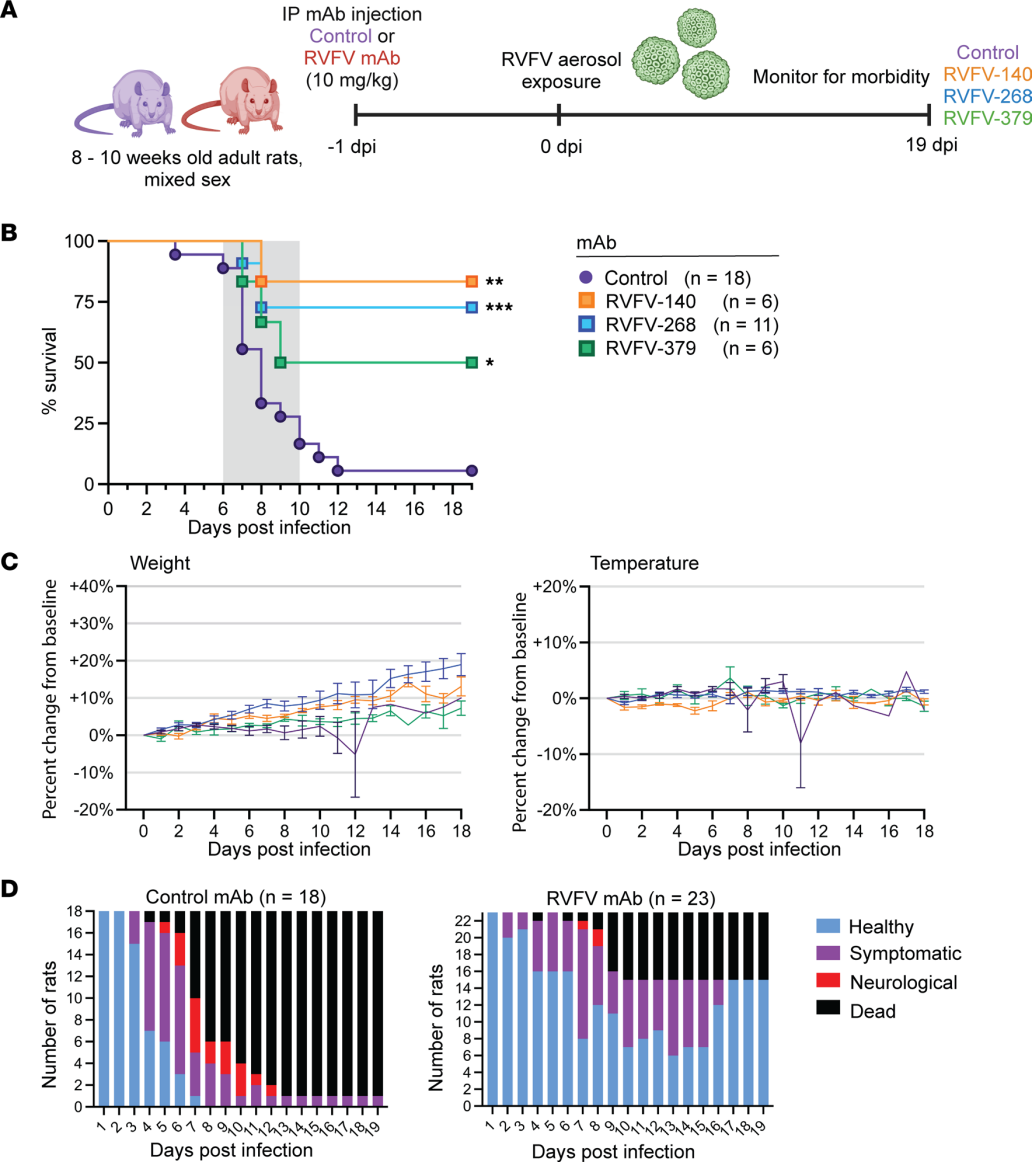
Human mAbs RVFV-140, RVFV-268, and RVFV-379 delivered prior to challenge significantly improve survival in rats exposed to pathogenic RVFV by inhalation. (**A**) Experimental design. Rats pretreated (*n* = 42 total) 24 hours prior to exposure with 10 mg/kg control (purple) or RVFV (blue) human mAbs by i.p. injection. Rats were exposed via whole-body aerosol to pathogenic RVFV (average 500 PFU) at 0 dpi and monitored for morbidity. (**B**) Survival from aerosol challenge after a total of 4 separate experiments. The gray box indicates the clinical window of neurologic disease (6–10 dpi). (**C**) The mean change in weight and temperature from baseline across all experiments (*n* = 5), grouped by mAb. Data are shown as mean ± SD. (**D**) Rats were monitored for morbidity using a clinical scoring system. Healthy rats displayed no clinical symptoms and had normal temperature. Symptomatic animals displayed fever, ruffled fur, perforin staining, or had weight loss ≥5% baseline weight. Rats with neurological symptoms displayed circling, head tremor, paralysis, and seizure. Rats meeting euthanasia criteria were humanely euthanized. Log-rank (Mantel-Cox) test was used to compare survival curves between RVFV mAb and control. *P < 0.05; **P < 0.01; ***P < 0.001.

We previously reported that prophylactic treatment using RVFV-268 or RVFV-140 significantly improved survival in a lethal hepatic disease model in mice using subcutaneous infection (18). To date, RVFV-379 has not been assessed in vivo, although it is considered potently neutralizing in vitro (Table 1) (18). Here, we tested RVFV-140, RVFV-268, and RVFV-379 as prophylaxis in an encephalitis model in adult rats. We administered 10 mg/kg of each mAb individually by i.p. injection 24 hours prior to whole-body aerosol exposure to pathogenic RVFV (ZH501 strain) (Figure 1A). As a control, we used an isotype-matched human mAb (DENV-2D22) targeting the envelope protein of the unrelated pathogen dengue virus. We exposed rats to an average of 500 PFU of RVFV (range, 200 PFU to 1.5 × 10^3^ PFU; ~4 × LD_50_) by aerosol across 4 experiments (totals, *n* = 18 control, *n* = 6 RVFV-140, *n* = 11 RVFV-268, *n* = 6 RVFV-379). Most rats pretreated with the control antibody succumbed to disease by 12 days postinfection (dpi) (17 of 18 mice died; 6% survival) while administration of RVFV-140 prior to challenge resulted in an 83% survival (5 of 6 mice survived); RVFV-268 demonstrated a 72% survival (8 of 11 mice survived); and RVFV-379 increased survival to 50% (3 of 6 mice survived) (Figure 1B). The 1 RVFV-140–pretreated rat that succumbed to disease at 8 dpi suffered fever, moved in small circles, was socially isolated, and displayed labored breathing. The control animals that succumbed had an average survival time of 8.5 days and displayed neurologic signs, which included circling, loss of muscle coordination, head tremors, paralysis, and seizures (Figure 1D). Control animals also showed substantial weight loss and a hypothermic/hyperthermic temperature fluctuation prior to meeting endpoint criteria (Figure 1C and Supplemental Figure 1; supplemental material available online with this article; https://doi.org/10.1172/jci.insight.180151DS1). None of the surviving anti-RVFV mAb–treated animals displayed neurologic signs throughout the study, and they gained or maintained weight (Figure 1C).

### Pretreatment with human mAb RVFV-268 improves viral control following exposure to RVFV.

**Figure 2:**
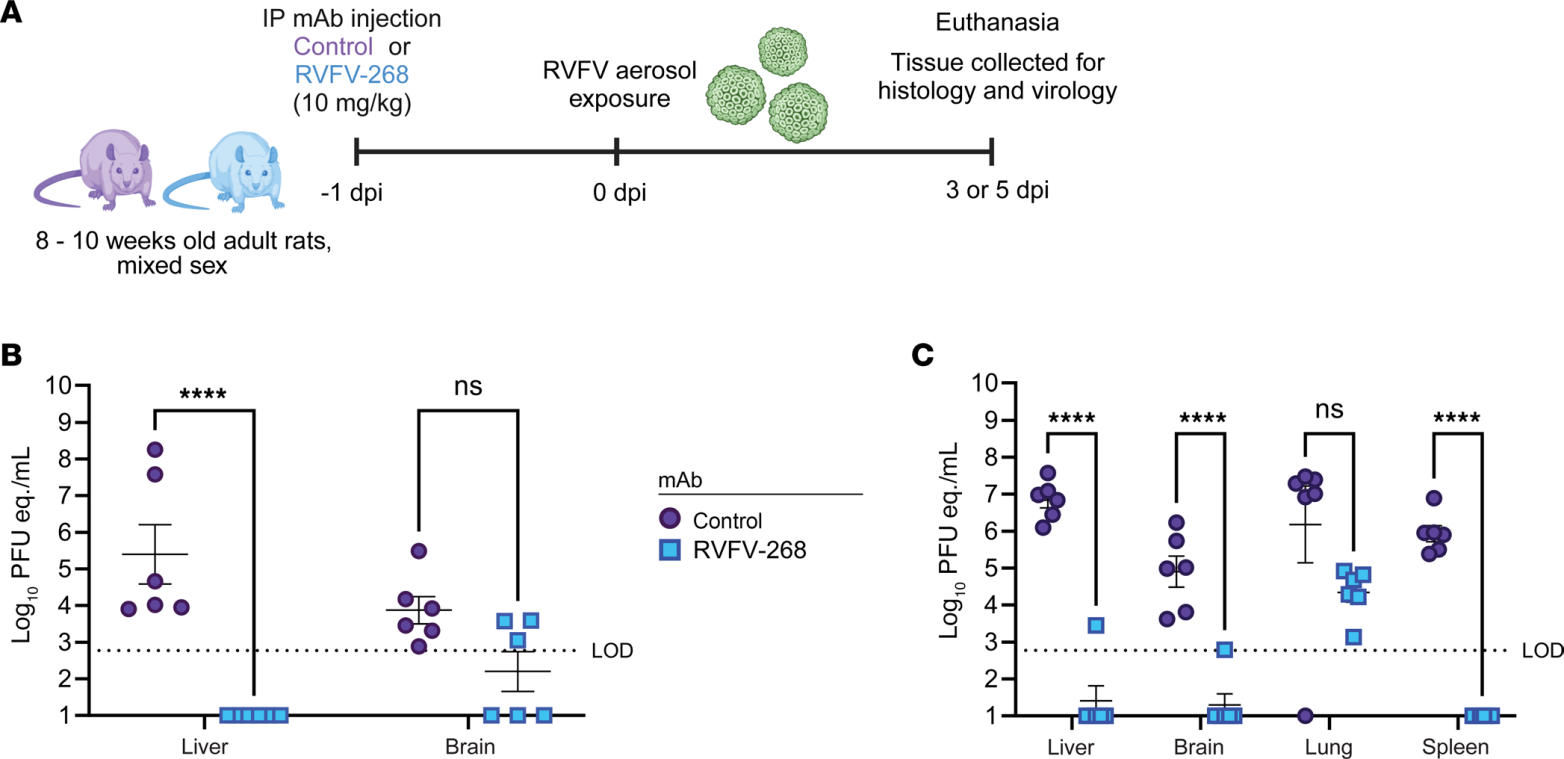
Human mAb RVFV-268 improves viral control in rats after exposure to aerosolized RVFV. (**A**) From 2 low-dose exposure experiments at 3 (**B**) or 5 (**C**) dpi, rats pretreated with control (*n* = 6) or RVFV-268 (*n* = 6) mAbs were euthanized, and tissue was collected from liver or brain (**B**) or liver, brain, lung, and spleen (**C**). Tissue homogenate was used to quantify viral titers by RT-qPCR. Data represent mean ± SEM. Statistics were determined using a 2-way ANOVA. ****P < 0.0001. LOD, limit of detection.

RVFV-268 is the most potently neutralizing antibody in vitro (IC_50_ value of <0.2 ng/mL) of the antibodies tested (Table 1). In a separate study using this antibody, we showed that RVFV-268 protected pregnant rats in a vertical transmission model of RVF. Both dams and their fetuses were completely protected from subcutaneous challenge with virulent RVFV based on lack of detection of viral RNA (vRNA) or infectious virus (20). Given the potency of RVFV-268, we focused on continued testing of it in a prechallenge format. To directly compare viral tissue burden between groups at matched time points, we pretreated 24 rats with either RVFV-268 (*n* = 11) or control (*n* = 12) antibody 24 hours prior to aerosol exposure as described above (presented dose 3 × 10^2^ PFU). We euthanized a subset of 6 animals per group at 3 dpi for a direct comparison of tissue titers (Figure 2A), while the remaining rats were monitored for morbidity. vRNA and infectious titers were measured by quantitative reverse transcription PCR (RT-qPCR) and viral plaque assay, respectively, on brain and liver tissue obtained from all animals. In both brains and livers from rats that received mAb RVFV-268, vRNA was at or below the limit of detection compared with the rats pretreated with control antibody, which contained moderate-to-high levels of vRNA (10^4^–10^8^ PFU equivalents per mL [PFU eq./mL]) (Figure 2B). We detected infectious virus in the liver of only 1 rat that was pretreated with RVFV-268, while infectious virus was not detected in the brains of rats pretreated with RVFV-268 (Supplemental Figure 2A).

**Figure 3:**
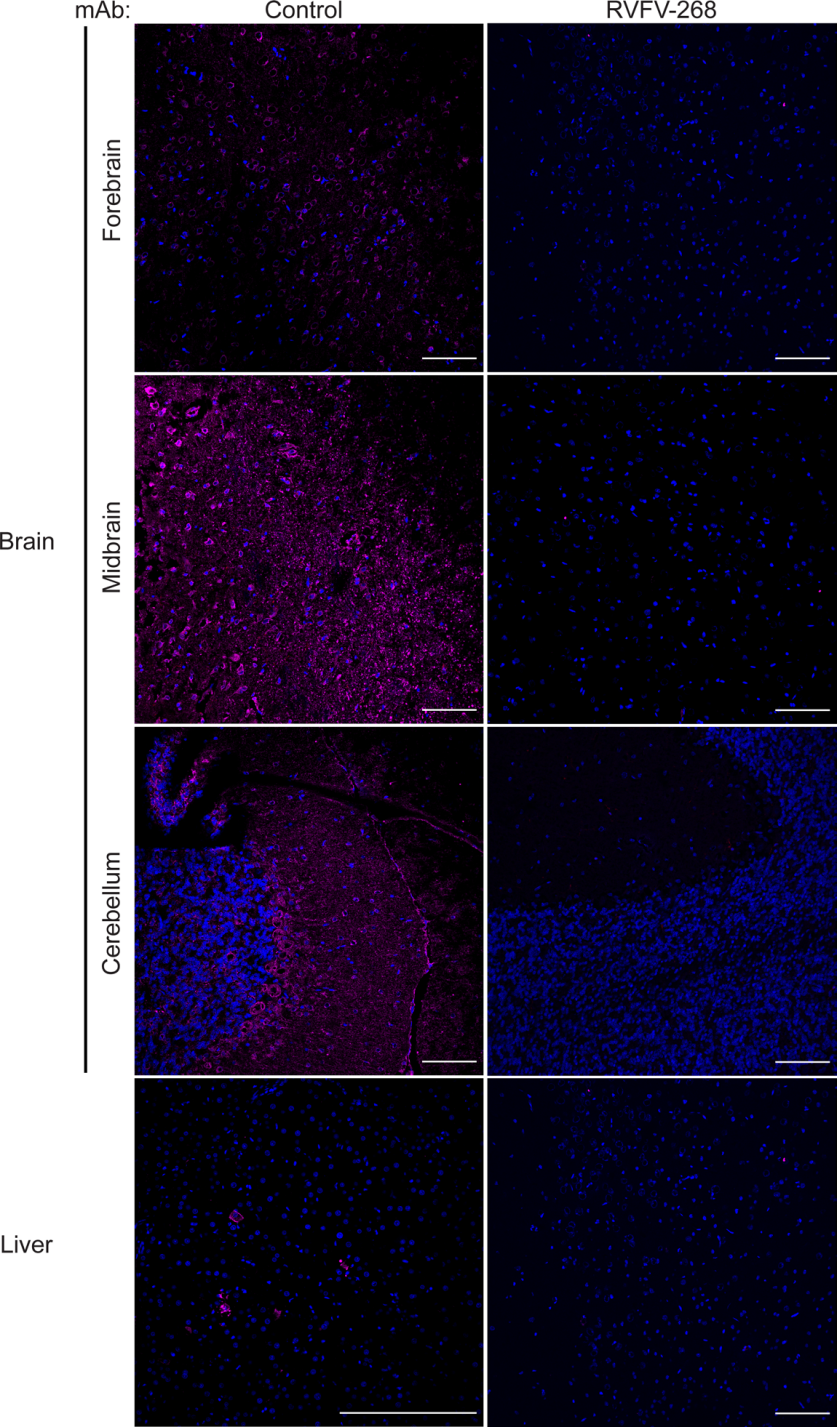
RVFV is less diffuse or not observed in the brains of rats pretreated with mAb RVFV-268. Immunofluorescence images of the forebrain, midbrain, cerebellum, and liver stained for nuclei (DAPI; blue) and RVFV nucleoprotein (magenta) from the brain or liver of rats pretreated with control antibody (left) or RVFV-268 (right). Original magnification, ×20. Scale bar: 100 μm (first 3 rows and bottom right); 200 μm (bottom left).

In a separate experiment, we conducted another planned euthanasia at 5 dpi instead of 3 dpi, as we expected that this time point would give better insight into viral burden immediately preceding neurologic disease. Twenty-four rats were pretreated RVFV-268 (*n* = 12) or control (*n* = 12) antibody at 10 mg/kg 24 hours prior to aerosol exposure to RVFV (presented dose 2 × 10^2^ PFU). At 5 dpi, we humanely euthanized 6 rats per cohort and obtained liver, brain, lung, and spleen tissues. The remaining rats (*n* = 12) were monitored for morbidity. At 5 dpi, control animals had high levels of vRNA in their liver (10^7^ PFU eq./mL) and lung (10^7^ PFU eq./mL), with intermediate levels in the brain (10^5^ PFU eq./mL) and spleen (10^6^ PFU eq/mL). In contrast, animals pretreated with RVFV-268 had undetectable or very low vRNA levels in the liver, brain, and spleen and modest (10^4^ PFU eq./mL) levels in the lung (Figure 2C). Animals pretreated with RVFV-268 did not have detectable infectious virus in their liver, brain, lung or spleen tissues at 5 dpi (Supplemental Figure 2B). Immunofluorescence microscopy of brain tissue obtained from animals at 5 dpi demonstrated that rats pretreated with mAb RVFV-268 had little (1 infected cell) to no viral antigen staining throughout the brain and none in the liver, while rats pretreated with the control antibody had viral antigen staining throughout the brain and in foci of the liver (Figure 3 and Supplemental Figures 3–5). These results correspond with the levels of vRNA and infectious virus detected in the tissues of these animals, supporting the finding that pretreatment with mAb RVFV-268 improves viral control following inhalational RVFV exposure.

## Discussion

RVF manifests a variety of disease outcomes in people. Approximately 50%–95% of individuals who contract RVFV will develop symptoms, which may include a self-limiting febrile illness with body aches (21). A portion of cases progress to more severe forms, including hemorrhagic fever, ocular disease, vertical transmission, or encephalitis. While development of RVF encephalitic disease in people is rare (1%–2%), analysis of patient data from the 2000 outbreak in the Arabian Peninsula showed a 53% case fatality rate among individuals with neurologic involvement (5). Survivors of neurologic manifestations of RVFV may experience long-term sequelae, including blindness, quadriparesis, and hemiparesis (2, 22).

The risk factors for the development of more severe manifestations remain poorly understood (21, 23). Some studies suggest that regular animal exposure, such as butchering, handling, living close to, and consuming animal products, is associated with increased likelihood of severe outcomes from RVFV infection (24–26). Importantly, it remains unknown what leads to the development of severe manifestations of RVFV, as host genetics, inoculum dose, and/or exposure route may be confounding factors. Testing vaccines and therapeutics for their efficacy against understudied disease outcomes, such as encephalitis, is a critical step to assessing their ability to prevent or treat the range of disease outcomes.

To date, there are no FDA-approved antiviral or antibody-based therapeutics for RVF. Given the range of disease outcomes resulting from RVFV infection, prophylactic or therapeutic treatment would ideally protect against the possible outcomes of RVFV infection, including encephalitis. Once a virus enters the brain, it becomes difficult to control spread, prevent damaging inflammation, and promote neuronal survival (27). Demonstrating therapeutic efficacy within the CNS is challenging, since the blood-brain barrier (BBB) tightly regulates access to the CNS. The BBB separates circulating blood from the brain, preventing brain uptake of most large molecules, including therapeutics. However, antibodies can cross the BBB in limited quantities (28). Some viruses antagonize the BBB function to promote viral entry, which may also allow for the increased infiltration of immune cells and antibodies (27). For RVFV, BBB breakdown is a late pathogenic event in rats, occurring after the virus is already in the brain but preceding death (12, 13). Therefore, providing protection early during disease is essential to controlling RVFV infection in the CNS.

The success of mAbs in providing protection against viral encephalitis has been shown in other arboviral diseases. For example, mAb therapy was efficacious in a nonhuman primate model of Venezuelan equine encephalitis virus infection following intravenous virus inoculation and also in mice exposed to aerosolized Eastern equine encephalitis virus (EEEV) when anti-EEEV mAb was administered i.p. (29, 30). For RVFV, neutralizing mAbs targeting the surface glycoproteins Gn or Gc are a known correlate of protection against disease (31). Passively transferring sera from vaccinated or surviving animals protects naive animals from peripheral challenge (32, 33). Prior isolation and characterization of anti-RVFV mAbs from survivors or vaccinated individuals demonstrated protection against lethal RVFV disease in mice (34–36). Defining the protective thresholds of serum neutralizing antibodies using defined doses of known neutralizing mAbs against precise disease manifestations may help inform vaccine performance and protective phenotypes. These studies provide a strong rationale for testing human mAbs in an aerosol model of RVFV infection.

We previously identified a panel of 20 human mAbs against RVFV isolated from B cells obtained from immunized or naturally infected individuals (18). These antibodies recognize diverse epitopes on viral surface glycoproteins Gn, Gc, or on the glycoprotein complex Gn-Gc. Of the 20 mAbs, RVFV-268 was identified as the most ultrapotent neutralizing mAb in vitro, with an IC_50_ of approximately 0.2 ng/mL. The epitope of mAb RVFV-268 was mapped to Gn domain A in a region that is accessible on the virion surface, and neutralization required bivalent binding by Fab_2_ or full IgG (20). RVFV-379 was identified as a potently neutralizing antibody, with an IC_50_ of 4.6 ng/mL. This mAb also mapped to domain A on Gn, directly competing for binding with RVFV-268. To date, RVFV-379 has not been tested in vivo. The third antibody tested in this study, RVFV-140, has moderate in vitro neutralization capacity at 13 ng/mL. In contrast to RVFV-268 and RVFV-279, RVFV-140 targets an unknown Gn-Gc–specific epitope and is thought to inhibit fusion. RVFV-140 provides sterilizing immunity in a lethal hepatic model of disease when administered prophylactically at 200 mg (18). Recently, it has been demonstrated that RVFV-268 and RVFV-140 are efficacious as a combination therapy at low doses (0.02 μg) in a lethal mouse model of infection (19). The contribution of Fc effector functions in protection has not been defined for these mAbs, and this topic warrants further investigation.

Here, we tested several mAbs in a preinfection format in an encephalitic disease model of RVF. Despite not providing 100% protection based on survival, prophylactic administration of human mAbs RVFV-140, RVFV-268, and RVFV-379 all significantly improved survival. Animals pretreated with mAb RVFV-268 demonstrated improved viral control at 3 and 5 days after exposure, and no infectious virus, except for in 1animal, was detected in brain or liver tissue. Surviving rats did not demonstrate acute neurologic symptoms through 21 days after infection. However, we did not perform long-term analysis of neurologic complications or behavioral changes following survival from RVFV infection, a topic that can be explored in future studies. Virological analysis of brain and liver tissue from survivors at 21 dpi detected vRNA at or below detectable limits, and infectious virus was not detected, supporting the finding that rats cleared infection following pretreatment with RVFV-268.

The results of this study complement a recent report showing efficacy of the human mAb RVFV-268 in a vertical transmission model of RVFV disease (20). Late-gestation pregnant dams (embryonic day 14) treated with 10 mg/kg of RVFV-268 2 hours prior to subcutaneous inoculation with pathogenic RVFV had no detectable virus in maternal or fetal tissue at 3 or 6 days after exposure. Dams and their offspring also were protected when RVFV-268 was administered up to 24 hours after exposure to pathogenic RVFV. Postchallenge mAb administration was likewise successful in mice against hepatic RVF disease when given 2 or 4 days after infection (18, 19). The current study did not assess if postexposure therapeutic administration of any of these antibodies affects encephalitic disease outcome in rats, and this question will be addressed in future studies.

While prophylactic i.p. administration of human mAbs provided sizable improvement to survival, we could not achieve 100% protection at a 10 mg/kg dose. Increasing the dose or altering the route of mAb administration may improve the efficacy of these mAbs. Intranasal-nose-to-brain administration has been effective in delivering IgG to the CNS in animal models (37). Additionally, others have demonstrated that antibody derivatives, including single-chain fragment variable (scFv) and single-domain antibodies are stable and small enough for inhalational (aerosol) or intranasal delivery (37). Current approaches to optimize uptake of serum antibodies to the brain include developing bispecific BBB-crossing antibodies (receptor-mediated transport) or using protein engineering to design antibodies with physiochemical, molecular, and binding properties optimized for transport across the BBB (28, 38). These delivery mechanisms should be explored to improve anti-RVFV mAb efficacy in the CNS. Finally, other antibody components, like Fc effector functions, may contribute to protective phenotypes that we are unable to achieve in these experiments. Optimization of these mAbs and the study design is warranted to further assess efficacy against encephalitic disease. This study is an important step in demonstrating efficacy of potent neutralizing mAbs against a very stringent aerosol infection model of RVFV neurological disease.

## Methods

### Sex as a biological variable.

Our study examined male and female rats, and we report similar findings for both sexes.

### Biosafety.

All experiments with RVFV were conducted in the Center for Vaccine Research and the Regional Biosafety Laboratory (RBL) at the University of Pittsburgh following all university and BSL-3 select agent regulations. The RBL is a registered BSL-3/ABSL-3 laboratory space with the Centers for Disease Control (CDC) and United States Department of Agriculture.

### Virus and cells.

The ZH501 strain of RVFV used in these experiments was originally obtained from Barry Miller (CDC, Atlanta, Georgia) and Stuart Nichol (CDC, Atlanta, Georgia) as described previously (12). Vero E6 cells (ATCC, CRL-1586) were used to propagate RVFV following standard cell culture conditions in Dulbecco’s modified Eagle’s medium (DMEM) containing 2% or 10% fetal bovine serum, 1% penicillin-streptomycin, and 1% L-glutamine. For quantitation, virus was measured using previously described methods of viral plaque assay and TaqMan q-RT-PCR (20).

### Human mAbs.

The human recombinant mAbs RVFV-140, RVFV-268, RVFV-379, and DENV-2D22 were generated as previously described (18, 19, 20). Each RVFV-specific antibody lot was tested against MP-12 for its ability to neutralize the virus and tested for binding against recombinant Gn as previous described (18, 19) before shipment. Antibodies were shipped overnight to the University of Pittsburgh and stored at –80°C until use. Antibodies were thawed and diluted in 1X PBS to 10 mg/kg to be administered i.p.

### Rat experiments.

Male and female Lewis (LEW/SsNHsd) and Sprague Dawley (Hsd:Sprague Dawley SD) rats were obtained from Envigo Laboratories at between 8 and 10 weeks of age. The data presented in this manuscript represent a compilation of samples from several independent experiments. Human mAbs (10 mg/kg) were administered i.p. 24 hours prior to viral challenge while under anesthesia during which temperature chips were also implanted subcutaneously in the dorsal area (Bio Medic Data Systems).

Rats were exposed to aerosols containing RVFV in a whole-body exposure chamber inside a class III biological safety cabinet located in the Aerobiology suite of the RBL. Exposures were controlled by the Aero3G management platform (Biaera Technologies) as previously described (39). Total air into and out of the exposure chamber was set to 19.5 liters per minute (lpm) to ensure 1 complete air change in the exposure chamber every 2 minutes. Aerosols were generated using a vibrating mesh nebulizer with airflow into the chamber at 7.5 lpm (39). Secondary air (12.0 lpm) included humidified air to achieve >80% relative humidity as previously described (40). An all-glass impinger (AGI; Ace Glass) with 10 mL of liquid media (DMEM containing glycerol and antifoam A) pulling vacuum at 6 lpm, ≤–7 psi was attached to the chamber and collected throughout the aerosol. Virus concentration in the aerosol was determined by plaque assay titer on the AGI contents (41). Inhaled virus dose was determined as the product of the aerosol concentration, the duration of the exposure (10 minutes), and minute volume of the rats. Minute volume was calculated based on weight using Guyton’s formula (42). The doses listed in these studies are the actual presented doses during the aerosol exposure. The presented doses were determined by sampling the air during the aerosol exposure and then performing plaque assay to calculate the presented dose.

For planned euthanasia experiments, a total of 24 rats were used, with 6 rats euthanized per cohort at 3 or 5 days to collect tissues. Rats in survival cohorts were euthanized as they met morbidity criteria. Rats typically reach euthanasia criteria by 6–10 dpi after aerosol exposure to this dose of RVFV. Blood was collected for serum analysis from the surviving mice. At the 3 and 5 dpi planned euthanasia, rats were anesthetized with isoflurane and blood was collected via cardiac puncture. The rats were then euthanized and necropsied to harvest brain, liver, lung, and spleen tissue, which was homogenized and titered using viral plaque assay Omni tissue homogenizer (Omni International).

### Immunofluorescence.

Brain and liver tissue collected from rats at 3 or 5 dpi was fixed in 4% paraformaldehyde for 24 hours, washed in 1X PBS, and submerged in the following sucrose concentrations for 24 hours each before storage at 4°C: 10%, 20%, 40%. Tissues were embedded in OCT (Fisher, 23-730-571), and brain sagittal regions were sliced at 10 μm; livers were sliced at 6 μm on a Cryostat (Cryostar NX70, Thermo Fisher Scientific). OCT was washed from slices using 1X PBS + 0.5% BSA (PBB). Tissue sections were permeabilized in 0.01% Triton X-100 detergent + 1X PBS for 15 minutes at room temperature. Tissue sections were blocked with 5% normal goat serum diluted in PBB for 45 minutes at room temperature, washed with PBB, and probed with mouse anti-RVFV N (1:200, BEI Resources, NR-43195) for 1 hours at room temperature. Following washes with PBB, the tissue sections were probed with goat anti-mouse FITC (1:500, Jackson Immuno, 115-095-003) secondary antibodies. Slides were washed with 1X PBS and stained with Hoechst for 30 seconds at room temperature before being mounted with Gelvatol. Fluorescent slides were imaged using a Nikon A1 confocal microscope at the Center for Biologic Imaging at the University of Pittsburgh. Images were processed using ImageJ (NIH).

### Statistics.

Statistical analyses were performed using GraphPad Prism software (10.0.2). For Figure 1, log-rank (Mantel-Cox) test was used to determine differences in survival between each human mAb pretreatment. For Figure 3, 2-way ANOVA was used to determine statistical significance between the groups at each time point with the 2 factors being mAb (RVFV-268 vs DENV-2D22) and tissue type. Tukey’s multiple-comparison test was also performed for comparison of the mean value for each tissue type within the treatment groups to each other. *P* values of less than 0.05 were considered significant.

### Study approval.

This work was approved by the University of Pittsburgh IACUC under protocol 20047334 and 23043040. All animal work was conducted in accordance with the recommendations in the *Guide for the Care and Use of Laboratory Animals* of the National Resource Council (National Academies Press, 2011). All animals were housed and fed in an Association for Assessment and Accreditation of Laboratory Animal Care–accredited facility. IACUC-approved euthanasia criteria were based on weight loss, fever, and morbidity.

### Data availability.

Values for all data points in graphs are reported in the Supporting Data Values file or are available from the corresponding author upon request.

## Author contributions

KAC, NSC, CMM, JEC, and ALH conceptualized and designed this study. KAC, RMH, JJM, CW, MM, and DSR contributed to data collection. KAC, NSC, JEC, and ALH analyzed and interpreted the results. JEC and ALH acquired funding for these studies. KAC and ALH drafted the original manuscript. All authors reviewed the results and approved the final version of the manuscript.

## Supplementary Material

Supplemental data

Supporting data values

## Figures and Tables

**Table 1:**
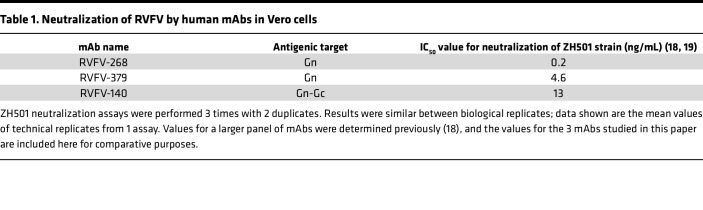
Neutralization of RVFV by human mAbs in Vero cells

## Version 1 08/01/2024

In-Press Preview

## Version 2 09/24/2024

Electronic publication
