# Supplementary material for: Potent neutralizing human monoclonal antibodies protect from Rift Valley fever encephalitis
Source: JCI Insight. 2024 Aug 1;9(18):e180151. doi: 10.1172/jci.insight.180151 (PMC11457859; doi:10.1172/jci.insight.180151)
Supplement: Supplemental data [file jciinsight-9-180151-s175.pdf]

A

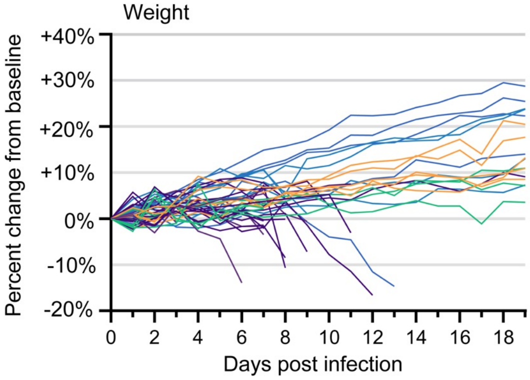

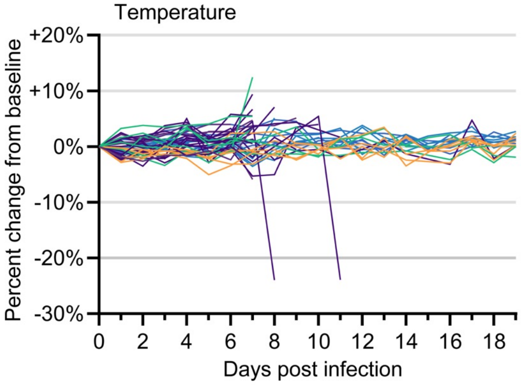

Supplemental Figure 1. Weight and temperature changes in rats following challenge with pathogenic RVFV by inhalation. (**A**) Change in weight and temperature from baseline across all experiments (4) for each individual rat shown per treatment group (n = 5).

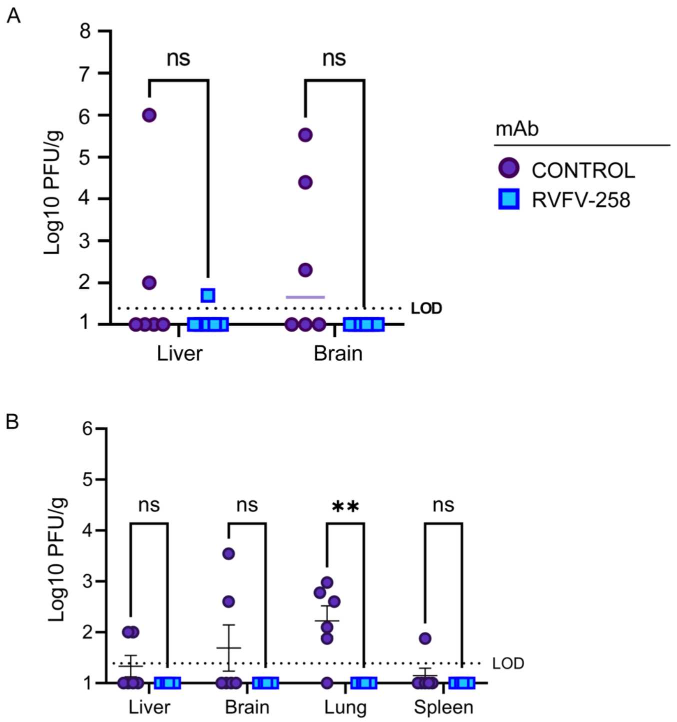

Supplemental Figure 2. MAb RVFV-268 decreases infectious viral titer in multiple tissue types following inhalational RVFV challenge.

Rats pre-treated with control (n = 6) or RVFV-268 (n = 6) mAbs were euthanized at 3 (**A**) or 5 (**B**) dpi. VPAs were performed to quantify infectious titer within the brain, liver and/or lung and spleen. Data represents mean ± SEM. Statistics were determined using a two-way analysis of variance (ANOVA). *P < 0.05; **P < 0.01; ***P < 0.001; ****P < 0.0001. ns, not significant.

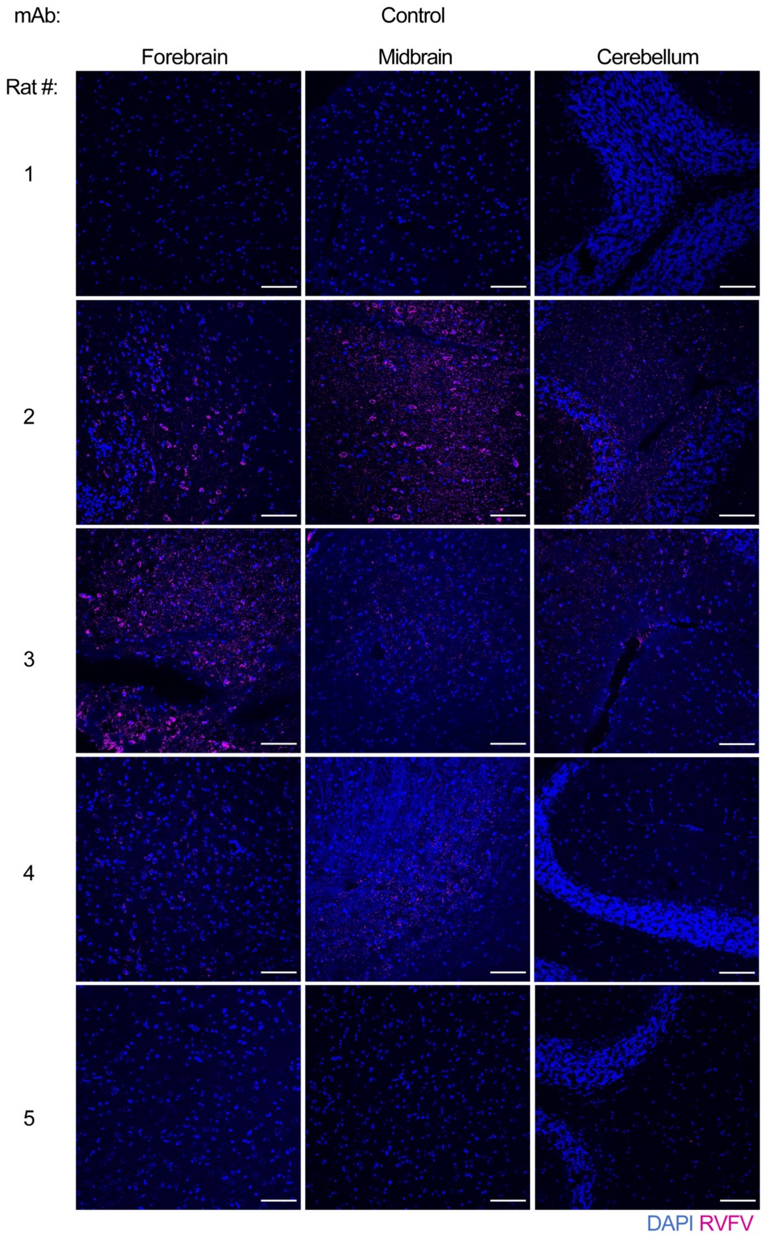

Supplemental Figure 3. Viral antigen staining is observed throughout the brain of rats pre-treated with control human mAb prior to challenge with pathogenic RVFV at 5 days post challenge. Immunofluorescent images of the forebrain, midbrain, cerebellum stained for nuclei (DAPI; blue) and RVFV N protein (magenta) from the brain of rats pre-treated with control antibody (n = 5). Each row represents tissue from a single rat. 20X magnification. Scale bar = 100 μm.

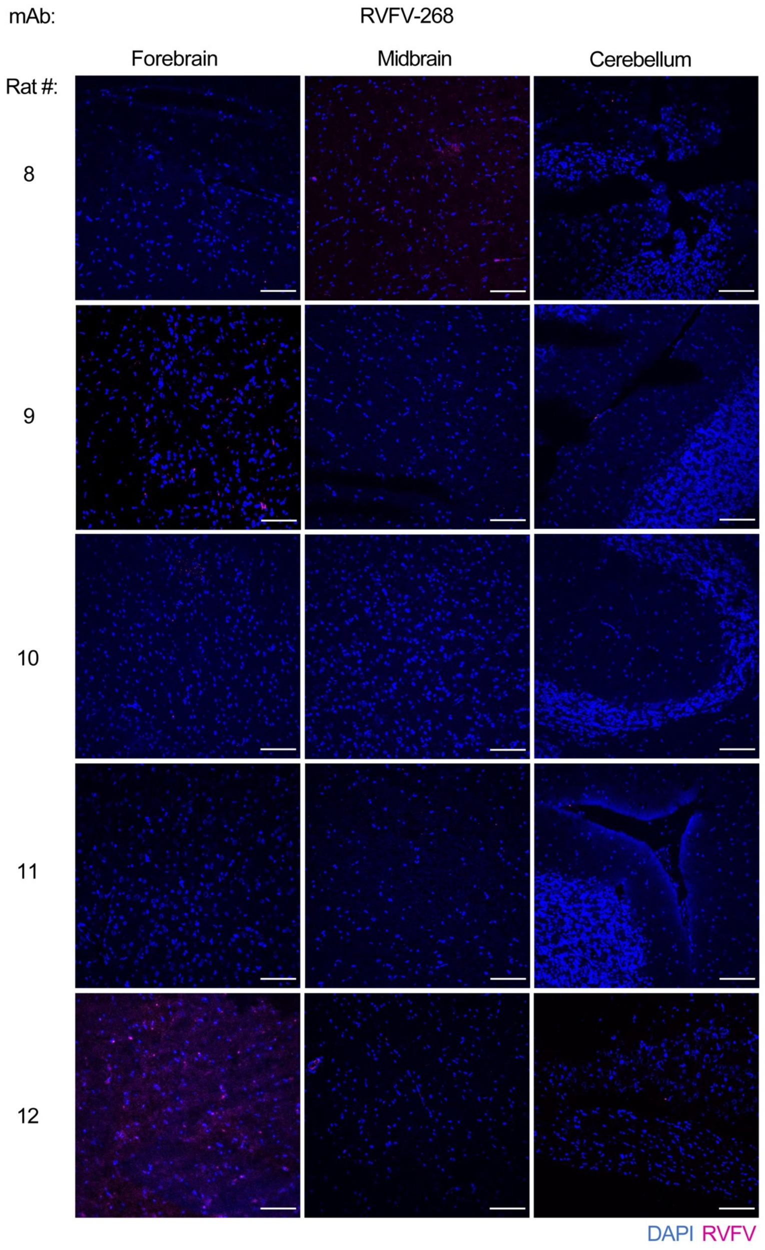

Supplemental Figure 4. Less viral antigen staining is observed throughout the brain of rats pre-treated with RVFV-268 prior to challenge with pathogenic RVFV at 5 days post challenge. Immunofluorescent images of the forebrain, midbrain, cerebellum stained for nuclei (DAPI; blue) and RVFV N protein (magenta) from the brain of rats pre-treated with RVFV-268 antibody (n = 5). Each row represents tissue from a single rat. 20X magnification. Scale bar = 100 μm.

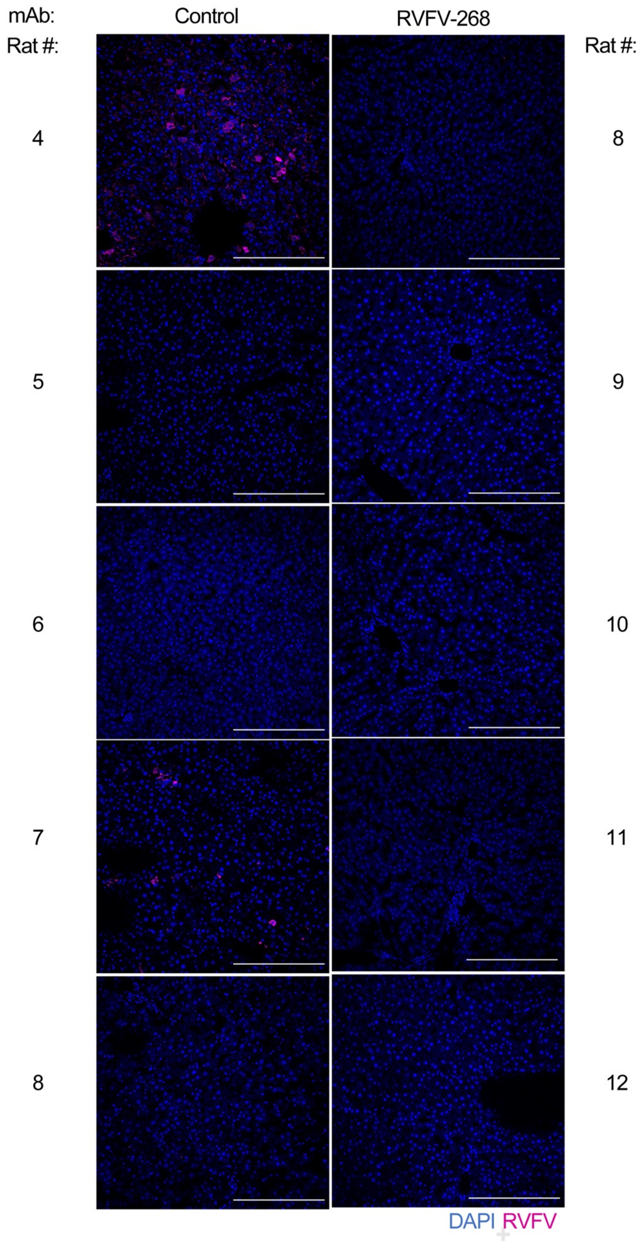

Supplemental Figure 5. Immunofluorescent microscopy in the liver of rats 5 dpi demonstrates less viral antigen staining after challenge in rats pre-treated with RVFV-268 compared to control. Immunofluorescence images of liver sections stained for nuclei (DAPI; blue) and RVFV nucleoprotein (magenta of rats pre-treated with control antibody (left column; rats 1-5) or RVFV-268 (right column; rats 8-12) and euthanized at 5 dpi. 20X magnification. Scale bar = 250 μm.
